# Radiographic Pearls in the Evaluation of an Extradural Thoracic Meningioma: A Case Report

**DOI:** 10.7759/cureus.1031

**Published:** 2017-02-15

**Authors:** Neil Haranhalli, Jonathan P Nakhla, Reza Yassari, Merritt D Kinon

**Affiliations:** 1 Department of Neurological Surgery, Montefiore Medical Center

**Keywords:** extradural spinal meningioma, dwi, lymphoma, neuroforaminal widening

## Abstract

Spinal meningiomas are the most common of adult spinal tumors. Spinal meningiomas account for up to 45% of all intradural spinal tumors in adults and up to 25% of all spinal tumors. While spinal meningiomas are traditionally classified as intradural lesions, up to 14% may have an extradural component. Preoperative evaluation and directed use of imaging techniques are key in these rare but observed cases, to accurately diagnose and direct therapy. In this report, the authors present a case of a 61-year-old female with an incidentally found, exclusively extradural thoracic meningioma treated with surgical resection, highlighting key radiographic pearls in the evaluation of these uncommon lesions.

## Introduction

Spinal meningiomas are the most common of adult spinal tumors. As the cells of origin for these tumors have been localized to the arachnoid cap cells in spinal dura mater, these tumors are most often classified as intradural extramedullary lesions. Spinal meningiomas account for up to 45% of all intradural spinal tumors in adults and up to 25% of all spinal tumors [1]. Treatment of spinal meningiomas is generally accepted to be gross total surgical resection; and therefore, accurate diagnosis of these tumors is key in the preoperative phase of treatment. While spinal meningiomas are traditionally classified as intradural lesions, up to 14% may have an extradural component; additionally, only a few case reports have been published of entirely extradural meningiomas. Preoperative evaluation and focused use of imaging techniques are key in these rare cases to accurately diagnose and direct therapy. Identifying the dural relationship with these tumors (i.e., extradural vs. intradural) is also important in surgical planning and for counseling the patient regarding risks and expectation of surgery. In this report, the authors present a rare case of an exclusively extradural thoracic meningioma, evaluated preoperatively through a number of imaging modalities, only to be confirmed on final surgical resection.

Informed consent was obtained from the patient for this study.

## Case presentation

A 61-year-old female with a history significant for chronic tobacco use, chronic obstructive pulmonary disease (COPD), and hypertension (HTN) originally presented to the emergency department at an outside hospital with complaints of two months of left upper extremity paresthesias. On exam, she was found to have a normal neurologic exam without any features concerning for myelopathy. She did, however, have mildly decreased sensation in her left third and fourth fingers and the palmar surface of the left hand as compared to the right. As part of her initial evaluation at the outside hospital, a cervical magnetic resonance imaging (MRI) scan was obtained which demonstrated mild degenerative cervical spondylosis worse at C6-7 and an upper thoracic spine mass causing severe canal stenosis. 

**Figure 1:**
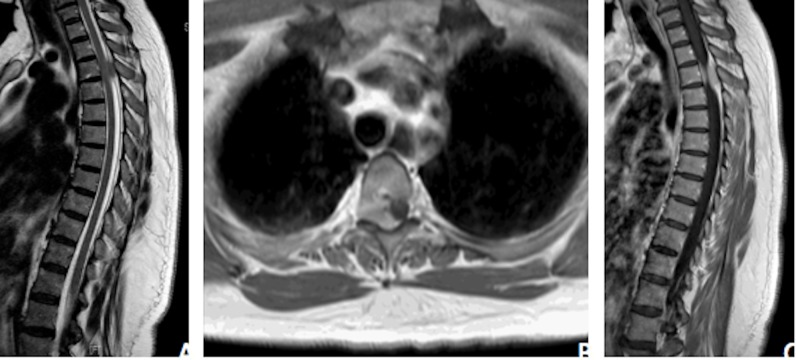
Preoperative Thoracic Spine MRI A. Preoperative sagittal T2 MRI demonstrating a thoracic lesion at T3 with resultant moderate compression of the spinal cord. B. Preoperative axial T1 post-gadolinium MRI demonstrating extension of the lesion into the right T3-4 neural foramina and causing leftward displacement of the cord. C. Preoperative sagittal T1 post-gadolinium MRI demonstrating homogenous enhancement of the thoracic lesion.

Initial evaluation of the thoracic lesion with a contrasted thoracic spine MRI showed a solid, homogenously enhancing right-sided extradural mass at the T3 level (Figure 1). The mass contacted the thecal sac along the lateral and ventrolateral surfaces, displacing the thecal sac to the left, causing cord compression with questionable abnormal cord signal. The mass also extended out of the right T3-4 neural foramen, widening it slightly.

**Figure 2:**
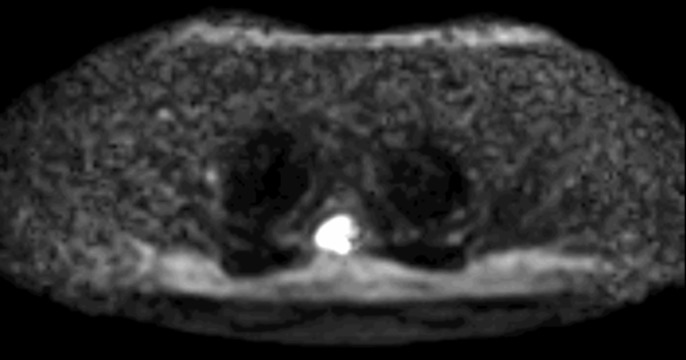
Preoperative DWI MRI Preoperative axial DWI MRI of the T3 thoracic lesion demonstrating marked diffusion restriction.

**Figure 3:**
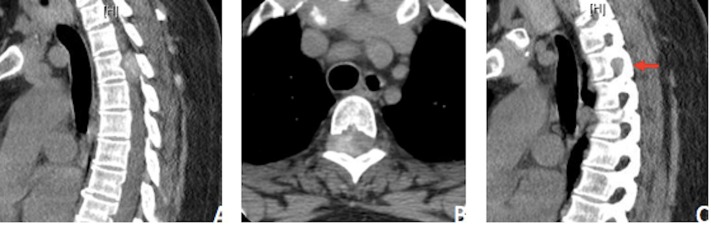
Preoperative Thoracic Spine CT A. Sagittal CT thoracic spine without contrast, demonstrating areas of patchy hyperdensity within the lesion suggestive of calcifications. B, C. Axial and sagittal CT thoracic spine without contrast showing bony remodeling and widening of the right T3-4 neural foramen.

The patient’s past medical history and the chest/abdomen/pelvis computed tomography (CT) were not concerning for metastatic disease; however, they did not rule out lymphoma or sarcoidosis. Consideration for lymphoma led to a repeat MRI with diffusion restriction, as prior studies have shown the utility of diffusion-weighted imaging (DWI) in the assessment of spinal lymphoma and other highly cellular tumors [2-3]. The tumor demonstrated marked restriction, supporting a possibility of lymphoma (Figure 2). With percutaneous biopsy felt to be too difficult given its location, involvement with the nerve root, and proximity to the spinal cord, surgical resection and decompression were pursued. The patient was started on a trial of steroids to see if the lesion changed in size; however, repeat MRI demonstrated no changes in the lesion. With the likelihood of lymphoma fairly low, the differential was further narrowed to a nerve sheath tumor or meningioma. Preoperative thoracic spine CT showed this mass to be hyperdense, potentially representing internal calcifications as well as bony remodeling of the T3-4 neural foramen suggestive of a slow growing process (Figure 3). Further review of the repeat contrasted thoracic spine MRI showed subtle signs of mildly enhancing dural tails, all suggestive of meningioma. 

Intraoperative findings were significant for an entirely extradural mass extending along an atrophic T3 nerve root. Intraoperatively there was an identifiable plane between the dura of the thecal sac and the capsule of the tumor, allowing for complete removal without the need for a durotomy or dural resection. The pathologic analysis confirmed World Health Organization (WHO) Grade I meningioma.

## Discussion

Spinal meningiomas represent approximately 12% of all meningiomas [1, 4]. They are typically classified as intradural extramedullary spinal tumors and makeup approximately 25% of this type of tumor. The peak incidence of spinal meningiomas is in the fifth and sixth decades with there being an almost ten to one predilection for females. In the spine, meningiomas are thought to arise from the denticulate ligaments and more than 95% are classified as WHO Grade I [3]. Their extradural component is most commonly related to extension from an intradural mass. Exclusively extradural meningiomas are seen in only five percent of spinal meningiomas [5]. Preoperative diagnosis of these tumors is most often based solely on imaging characteristics as seen on contrast-enhanced MRI. 

This case depicts an uncommon presentation of a common tumor and highlights key radiographic analyses that are useful in evaluating these rare presentations. Given the patient's presenting symptoms, a thoracic spinal lesion in this patient was an unexpected finding. Intermittent, unilateral paresthesias of the hand and forearm most often suggest pathology in either the cervical spine, brachial plexus, or peripheral nerves. She did have mild cervical spondylosis most significant at C6-7 which correlated with her symptoms and improved with a course of oral steroids; however, the thoracic lesion was an incidental finding, necessitating further workup.

While an intramedullary process could be ruled out on review of the T2-weighted and T1 contrast-enhanced MRI (Figure 1), which revealed that there was no expansion of the spinal cord nor any significant abnormal cord signal, initial evaluations did not point to a classical spinal meningioma. An intradural process, as most commonly seen with spinal meningiomas, was considered to be unlikely on review of the sagittal MRI sequences, which demonstrated tapering of cerebrospinal fluid (CSF) signal around the cord, cranially and caudally to the lesion, suggesting external compression of the thecal sac. The expansion of the neural foramen seen on CT was further evidence against a classic intradural meningioma. These findings were more suggestive of a nerve sheath tumor such as a neuroma or schwannoma. To the authors’ knowledge, this is the first such radiographic finding reported in the literature. Finally, assessment of the lesion with DWI revealed highly restricted diffusion within the lesion, suggestive of lymphoma. While the DWI characteristics of spinal meningiomas are not well described in the literature, these findings directed treatment in this case to a trial of steroids for suspected lymphoma with aims of a surgery-sparing cure. The diagnostic difficulties seen in this case shed light on the continued limitations of the traditionally image-based diagnosis of spinal meningiomas. Keeping a wide differential diagnosis and exhausting multiple imaging modalities, while important, has shown to still leave some questions unanswered. While extradural spinal meningiomas are an uncommon entity, it remains an important consideration in cases with poorly identified radiographic findings.

## Conclusions

This report presents an uncommon finding of an exclusively extradural thoracic meningioma. The radiographic findings in this case highlight the need for critical evaluation of such lesions. While WHO Grade I spinal meningiomas are most commonly intradural, exclusively extradural lesions are also seen, and as this case depicts, an accurate and detailed utilization of radiographics can improve the evaluation and eventual cure of these tumors. We hope that the combination of radiographic findings in this case serves as a guide for others who encounter this entity so as to consider meningiomas higher on the differential and offer patients a curable surgery without delay.
